# From Transcriptomics, Metabolomics to Functional Studies: Extracellular ATP Induces TGF-β-Like Epithelial Mesenchymal Transition in Lung Cancer Cells

**DOI:** 10.3389/fonc.2022.912065

**Published:** 2022-06-30

**Authors:** Maria Evers, Jingwen Song, Pratik Shriwas, Harrison S. Greenbaum, Xiaozhuo Chen

**Affiliations:** ^1^ Honors Tutorial College, Ohio University, Athens, OH, United States; ^2^ The Ben May Department for Cancer Research, University of Chicago, Chicago, IL, United States; ^3^ Department of Biological Sciences, Ohio University, Athens, OH, United States; ^4^ The Molecular and Cellular Biology Program, Ohio University, Athens, OH, United States; ^5^ The Edison Biotechnology Institute, Ohio University, Athens, OH, United States; ^6^ Department of Biomedical Sciences, The Heritage College of Osteopathic Medicine, Ohio University, Athens, OH, United States

**Keywords:** EMT, invasion, RNA sequencing, cancer metabolism, ROS, cytoskeleton remodeling, heatmap, TCA cycle

## Abstract

We and others previously showed that extracellular ATP (eATP) is implicated in epithelial mesenchymal transition (EMT). However, the mechanisms by which eATP induces EMT and ATP’s relationship to TGF-β, a well-known EMT inducer, are largely unclear. Also, eATP-induced EMT has never been studied at transcriptomic and metabolomics levels. Based on our previous studies, we hypothesized that eATP acts as a specific inducer and regulator of EMT at all levels in cancer cells. RNAseq and metabolomics analyses were performed on human non-small cell lung cancer (NSCLC) A549 cells treated with either eATP or TGF-β. Bio-functional assays, such as invasion, intracellular ATP, cell proliferation, cytoskeleton remodeling, and others were conducted in NSCLC A549 and H1299 cells to validate changes observed from RNAseq and metabolomics studies. In the RNAseq study, eATP significantly enriched expressions of genes involved in EMT similarly to TGF-β after 2 and 6 hours of treatment. Samples treated with eATP for 2 hours share 131 upregulated EMT genes with those of TGF-β treated samples, and 42 genes at 6 hours treatment. Eleven genes, with known or unknown functions in EMT, are significantly upregulated by both inducers at both time points, have been identified. *BLOC1S6*, one of the 11 genes, was selected for further study. eATP induced numerous EMT-related changes in metabolic pathways, including cytoskeleton rearrangement, glycolysis, glutaminolysis, ROS, and individual metabolic changes similar to those induced by TGF-β. Functional bioassays verified the findings from RNAseq and metabolomics that eATP EMT-like changes in A549 and H1299 cells similarly to TGF-β. *BLOC1S6* was found to be implicated in EMT. In these studies, eATP-induced EMT, at all levels examined, is similar but non-identical to that induced by TGF-β, and functions in such a way that exogenous addition of TGF-β is unnecessary for the induction. The study of *BLOC1S6* further verified its potential roles in EMT and the RNAseq analysis results. All these strongly indicate that eATP is a multi-functional and multi-locational inducer and regulator of EMT, changing our thinking on how EMT is induced and regulated and pointing to new directions for inhibiting EMT in cancer.

## Introduction

Metastasis is associated with up to 90% of all cancer-related death (1) but is also one of the least understood processes in cancer. Metastasis starts with a process known as epithelial to mesenchymal transition (EMT), in which a group of epithelial (E) related genes in cancer cells are downregulated and phenotypes suppressed while some mesenchymal (M) genes are upregulated and M phenotypes are expressed (2, 3), in preparation for subsequent invasion and metastasis. EMT also occurs in normal processes such as early embryonic development when cell migration and cell differentiation are required (3). EMT in cancer cells appear to be a process that mimics EMT in normal cells to achieve their own purpose of increased migration and survival.

EMT itself is a complicated and partially understood process that involves various sub-steps with each corresponding change in cell signaling, gene expression, metabolism, cytoskeleton remodeling, motility, and cell functions (4, 5). In addition, cells of different cancer types have been shown to respond to various kinds of tumor microenvironmental (TME) stimuli and express somewhat different sets of genes to reach a general state termed partial EMT (6–8). In a partial EMT state, which happens in all cancer cells examined upon EMT induction, cancer cells of different cancer types upregulate an incomplete and somewhat different set of mesenchymal genes and downregulate an incomplete and different set of epithelial genes depending upon cancer types, induction conditions, and TME cues (6–8). All of these indicate the plasticity and heterogeneity of EMT in cancer (3). Exactly how EMT is induced and regulated in cancer is far from fully understood, but TGF-β has long been recognized as a major EMT inducer and regulator (9, 10). TGF-β binds and activates cell membrane-associated TGF-β receptors. The activation of TGF-β receptors results in cascades of intracellular signaling, leading to EMT-related gene expression, metabolic reprograming, and phenotypic changes (11, 12). One of the TGF-β signaling pathways and induced processes described in cancer cells is the exocytosis of cytosolic ATP-containing vesicles, releasing ATP to the extracellular environment and creating an autocrine/paracrine signaling loop through extracellular binding and activating of purinergic receptors (PR) located on plasma membrane of cancer cells (13, 14). PR signaling by ATP is vitally important for inducing EMT (15). However, ATP-PR signaling is only one of the several signaling pathways induced by TGF-β, and it remained unclear if extracellular ATP alone is sufficient to induce EMT at levels from transcription, metabolism, to other functionalities.

Ample evidence strongly suggests that extracellular ATP (eATP) plays very important roles in EMT and metastasis. First, intratumoral eATP (ieATP) in TME has been found to be in a concentration range of 10^3^ to 10^4^ times higher than those in normal tissues (16–19), between ~200-600 μM. Previously, we reported that eATP, at the reported intratumoral concentrations, was internalized by macropinocytosis, a special form of endocytosis (20–22), both *in vitro* and *in vivo* in cancer cells of various cancer types (23–26). We also reported that internalized ATP greatly elevates intracellular ATP levels, which in turn increases the cancer cell proliferation rate and resistance to anticancer drugs, including those target drugs that compete with ATP for the ATP binding site of the protein targets (27, 28). More recently, we have reported the observation of a wide variety of eATP-induced EMT related phenotypic changes, including cell detachment, cell migration and invasion, morphologic and motility changes including loss of cell polarity, cytoskeleton remodeling, and protein expression alterations in human NSCLC A549 cells (29). All these changes cover almost entire spectrum of distinctive EMT features characterized *in vitro* (3), indicating that eATP induces EMT. In addition, ATP is a transcription cofactor involved in various steps of the transcription process (30–34). Extracellular ATP is also a danger signal in both bacteria (35) and animal cells (36), including cancer cells, informing cells to leave its present location for a safer place; it functions as a warning signal for migration, invasion, and metastasis in cancer.

However, if/how much eATP induces corresponding changes at either the gene expression or metabolic level during EMT process have never been systematically investigated, let alone investigated together. In addition, the similarities and differences between eATP and TGF-β in EMT induction at these levels were never compared.

In this study, we hypothesized that eATP functions as key master inducer and regulator for EMT similar to TGF-β, at all biological levels in cancer cells. Thus, we combined RNA sequencing (37, 38) with metabolomics analyses to test this hypothesis primarily in A549 cells and verified these results with various functional studies including studies of a novel gene identified from the RNAseq study. These studies were included because EMT is characterized by the expression of specific genes with subsequent metabolic reprogramming in cancer cells (3). These metabolites are the end products of EMT induction and correspond to the phenotypic changes associated with EMT. We then performed various bio-functional assays to characterize cellular and functional changes for supporting, confirming, and validating eATP-induced and -regulated the onset EMT revealed by RNAseq and metabolomics analyses. These assays demonstrate that EMT-related gene expression leads to EMT-related phenotypic changes. We used TGF-β as a known EMT inducer and TGF-β treated NSCLC cell lines as cell controls for comparison. In addition, we evaluated the importance and contribution of eATP-enhanced intracellular ATP concentrations in eATP-induced EMT. As a final step, we selected a gene identified from the RNAseq analysis, and studied its potential roles in EMT. The results of these studies show significant and independent contributions of eATP to EMT, and possibly the entire metastatic process.

## Materials and Methods

### Chemicals, Proteins, and Antibodies

Cell culture reagents were purchased from VWR. ATP was purchased from Sigma-Aldrich. TGF- β1 protein was from Cell Signaling. Pan-neutralizing antibody against TGF-β ligands was from Cell Signaling.

### Cell Lines and Cell Culture

Human non-small cell lung cancer (NSCLC) cell lines A549 and H1299 were purchased from ATCC. A549 and H1299 cells were cultured in Dulbecco’s Modified Eagle Medium (DMEM) as previously described (25–29).

We chose human lung cancer A549 and H1299 cells for the study because lung cancer has been our lab’s major cancer target and these two NSCLC cell lines have been used in our and other labs frequently before (24–27).

### RNA Sequencing

#### Sample Preparation

A549 cells were treated by ATP or TGF-β for 2 or 6 hrs because we were primarily interested in focusing on and catching early phase gene expression which is responsible for the induction of EMT. The timing was also based on the previous study, which revealed that EMT-related phenotypic changes occur around 2-6 hours post-induction (29). In addition, preliminary data showed a pattern of several EMT-TF levels peaking 6 hours post treatment (unpublished observation). After treatment, RNA was isolated from cells using an RNA Isolation kit (Thermo Fisher) following manufacturer’s instructions. Genomic DNA was removed from the samples using a genomic DNA removal kit (Thermo Fisher). RNA samples were eluted in DEPC treated water (Thermo Fisher). RNA Integrity number values were measured by the Ohio University Genomics Facility to ensure quality of the RNA before samples were shipped to LC Sciences in Houston, TX, for a total polyA RNA sequencing.

#### RNA Sequencing Data Analysis

A complete transcriptome was assembled after the sequencing and the primary analysis of the transcriptome provided by the company were further analyzed to identify gene expression changes associated with EMT induction. Analyses were performed to identify gene sets that were enriched in ATP and TGF-β treated cells, and gene sets were considered significantly enriched if their false discovery rate (FDR) q value was < 0.10, which is 15% less than the value recommended by the GSEA user guide (< 0.25). Heat map genes were identified from GSEA enrichments (39–41). Identified genes expression levels were averaged between samples, gene names were converted from ensemble transcript ID to universal gene symbol using the DAVID database (42, 43), and top hits were visualized utilizing Morpheus heatmapping software form the Broad Institute (https://software.broadinstitute.org/morpheus).

### Metabolomics Analysis

Sample preparation: Treatment times of 2, 6, and 12 hours were chosen for the reasons of matching with the RNAseq conditions (2 and 6 hr) and including a condition for later phase of EMT (12hr). A549 cells were treated as follows: no treatment/control,.5 mM eATP, and 10 ng/mL TGF-β. Cell culture metabolism was stopped by briefly incubating in ddH_2_O, metabolites were extracted by 80% ice cold methanol, and samples were sonicated and centrifuged at 4°C. Metabolites are present in the supernatant, which was removed and freeze dried (44). Samples were stored in -80°C and analyzed at the Ohio State University Campus chemical instrumentation center (CCIC).

Metabolomics data analysis: Metabolomics study was completed by quantitative untargeted LC-MS utilizing Q-TOF 6545 mass spectrometer connected to an Agilent 1290 UHPLC system with a Poroshell 120 SB-C18 (2 × 100 mm, 2.7-μm particle size) column. Metabolomics data was generated and received from CCIC. Masshunter software (Agilent Technology) was used for acquiring data and peaks were integrated using Progenesis (Agilent Technology). Compounds were identified with XCMS as well as Metaboanalyst 5.0 software. Peak areas were normalized using internal standards and were subjected to relative quantification analyses with control (no treatment).

### Invasion Assays

Cancer cell invasion assays, which are based on cancer cells penetrating a porous filter covered with a layer of Matrigel in an inner well (the well in another outer well) Transwell system, were performed as previously described with more extensive dose-dependent studies (29). The invading cancer cells have to “eat through” the Matrigel layer to reach the other side of the filter to be stained and then counted. The cells were incubated under different conditions (treatments applied to both the upper and lower chambers. After 20 hours incubation at 37°C, invaded cells to the bottom (opposite side) of the polycarbonate membrane of the upper insert were fixed with 4% paraformaldehyde and stained with crystal violet, and visually counted from six representative visual fields per well in experimental triplicate using compound light microscopy (200X magnification), and then averaged. The TGF-β concentrations used in these assays were the commonly reported ones in the literature.

### Fluorescence Microscopy of eATP Treated Cancer Cells

To observe morphological changes of filopodia-like protrusions, fluorescence microscopy was performed as previously described with an extensive time-dependent study (29). Briefly, F-actin (filamentous-actin) of cells were stained with Fluorescent Phallotoxins. A549 or H1299 cells were seeded overnight on glass coverslips placed in 24-well plates, then treated with or without ATP or TGF-β for various time periods (2, 6, and 12 hours). Stained cells were examined and photographed using a Fluor Motorized DIC Polarization Phase Contrast Microscope (Zeiss AXIO Observer) at 200× magnification.

### ATP Assay

ATP assays, which are based on measurement of light intensity generated from a biochemical reaction that uses ATP in cell lysates as energy source, were performed as previously described (25, 26, 29).

### Cell Proliferation/Viability Assay

Cell viability was measured using a Resazurin Assay. A549 cells were treated as follows: no treatment/control,.25 μg/ml TGF-β antibody,.50 μg/ml TGF-β antibody,.75 μg/ml TGF-β antibody,.25 μg/ml TGF-β antibody +.5 mM ATP,.50 μg/ml TGF-β antibody +.5 mM ATP,.75 μg/ml TGF-β antibody +.5 mM ATP, and.5 mM ATP for 24 hours. Media was aspirated from cells and replaced with fresh media containing 0.0045% of resazurin dye. Cells were incubated at 37°C for 10 minutes and the plate was read with a fluorescence microplate reader (Citation 3, BioTek) with excitation at 570 nm and emission at 590 nm.

### Western Blot Analysis

Proteins were isolated from cells treated with no treatment,.5 mM ATP for hours or 6 hours, or 10 ng/ml TGF-β for 2 hours or 6 hours. Proteins were analyzed with western blots using appropriate primary rabbit anti-human antibodies all purchased from Cell signaling Technologies: E-cadherin (# 3195), Snail (#3879), Vimentin (# 5741), MMP-1 (#54376), MMP-3 (# 14351), MMP-9 (# 2270), Claudin-2 (# 48120), and NF-κBp65 (# 4764). Secondary antibody staining was completed with anti-rabbit IgG, HRP-linked antibody (Goat, 1:1000, CST, #7074). Cofilin (D3F9) XP^®^ Rabbit mAb (#5175) was used as a protein loading control. The signals were detected with Super Signal West Pico Chemiluminescent substrate (Thermo Fisher Scientific) and was developed using (Odyssey Fc 2800, LI-COR Biosciences). Intensities of protein bands were quantified by the corresponding Odyssey Fc software used to develop blots.

### Study of Gene BLOC1S6 and Its Functions

To test the functional relevance of consistently upregulated genes identified from RNAseq analysis, we selected *BLOC1S6* as a study target for the following reasons. It is a gene relatively unknown for its functions, particularly totally unknown in EMT. In addition, it is a gene that its mRNA expression levels are inversely proportional to the survival of lung cancer patients with a large survival margin between low and high *BLOC1S6* expressors (45). This comparison of tumor samples from close to one thousand human lung cancer patients strongly implies that *BLOC1S6* expression levels significantly affect the survival of lung cancer patients.

The reasons for focusing on *BLOC1S6* also include (a) *BLOC1S6* is one of the eleven genes that were found to be consistently upregulated by both eATP and TGF-β at both detecting times (**Table 1**). Thus, BLOC1S6 is likely to play important roles in EMT. (b). *BLOC1S6* is one of several genes among the 11 genes that has not been studied in the fields of cancer and EMT. Thus, studying *BLOC1S6* would be more impactful than studying other well-known genes on the list such as *Sox8*, *BMP6*, and *MMP10* (**Table 1**). (c) In addition, earlier studies by others suggested that the protein BLOC1S6 is involved in intracellular vesicle trafficking (46), a process similar to macropinocytosis which is heavily used in eATP internalization as we observed (20–22, 25–29). This further links BLOC1S6 with eATP functions and EMT.

**Table 1:** Comparison of common genes significantly up- and down-regulated by eATP and TGF-β.

Upregulated Conserved Genes					
		log2(FC) values			
Gene Symbol	Gene Name	2 hr ATP	6 hr ATP	2 hr TGF-β	6 hr TGF-β
SOX8	SRY-Box Transcription Factor 8	2.01	2. 94	2.72	2.91
STC1	Stanniocalcin-1	3.41	1.29	1.48	1.39
BMP6	Bore morphogenetic protein 6	1.70	1.27	1.17	1.77
GJB3	Gap Junction Protein Beta 3	2.12	1. 73	1.64	1.40
MMP10	Matrix Metallopeptidase 10	1.36	1. 87	1.10	2.51
BLOC1S6	Biogenesis Of Lysosomal Organelles Complex 1 Subunit 6	1.40	1.27	1.51	1.39
ATP6V1G2-DDX39B	ATP6V1G2-DDX39B readthrough	1.57	2. 31	2.04	2.43
ILIA	Interleukin 1 Alpha	1.39	1. 06	3.06	3.95
LRRC38	Leucine Rich Repeat Containing 38	1.78	1.20	2.59	2.49
PLXNA4	Plexin A4	1.61	1. 10	3.40	3.61
SYTL3	Synaptotagmin Like 3	1.23	1. 04	1.03	1.12
**Downregulated Conserved Genes**					
		**logZ(FC) values**			
**Gene Symbol**	**Gene Name**	**2 hr ATP**	**6 hr ATP**	**2 hr TGF-β**	**6 hr TGF-β**
FOS	c-Fos	-4 .20	-1. 05	-3.21	-3.32
VAV3	Vav Guanine Nucleotide Exchange Factor 3	-1.07	-1. 11	-1.50	-1.94
ANKS4B	Ankyrin Repeat And Sterile Alpha Motif Domain Containing 4B	-1.61	-1.03	-1.55	-2.26
LINC00488	Long Intergenic Non-Protein Coding RNA 488	-1.21	-1.08	-2.02	-2.32
LINCD1783	Long Intergenic Non-Protein Coding RNA 1783	-1.92	-1.44	-1.91	-1.71
DAG1	Dystroglycan 1	-1.74	-1. 74	-2.40	-1.90
AGPAT3	l-Acylglycerol-3-Phosphate 0-Acyltransf erase 3	-1.23	-1. 31	-1.24	-1.20

Gene expression values are expressed in Log2 fold changes (Log2fc). All those values shown in **Table 1** are statistically significantly different from those of the untreated controls (Log2fc = 0). Positive and negative values represent gene upregulations and gene downregulations, respectively.

An siRNA study was conducted to knockdown (KD) the expression of BLOC1S6. Sequences of *BLOC1S6* siRNA is 5’-ATACGAGGTTTCATTGTTTAA -3’.

The transfection of the siRNA into A549 cells was performed as previously described (29). Functional assays such as cell proliferation, invasion drug resistance, and soft agar anchor-independent colony formation assay (47), were conducted to assess the potential roles of BLOC1S6 in EMT.

### Data Analysis

Each experimental condition will be performed in at least triplicates and repeated at least once. Results were reported as mean ± standard deviation. The statistical difference, or difference between control and treatment groups, was analyzed using Student’s t-test or one-way ANOVA. P<0.05 was considered statistically significant.

## Results

### RNA Sequencing Reveals That eATP Induces Gene Expression Similar but Not Identical to Those Induced by TGF-β

**Figure 1:**
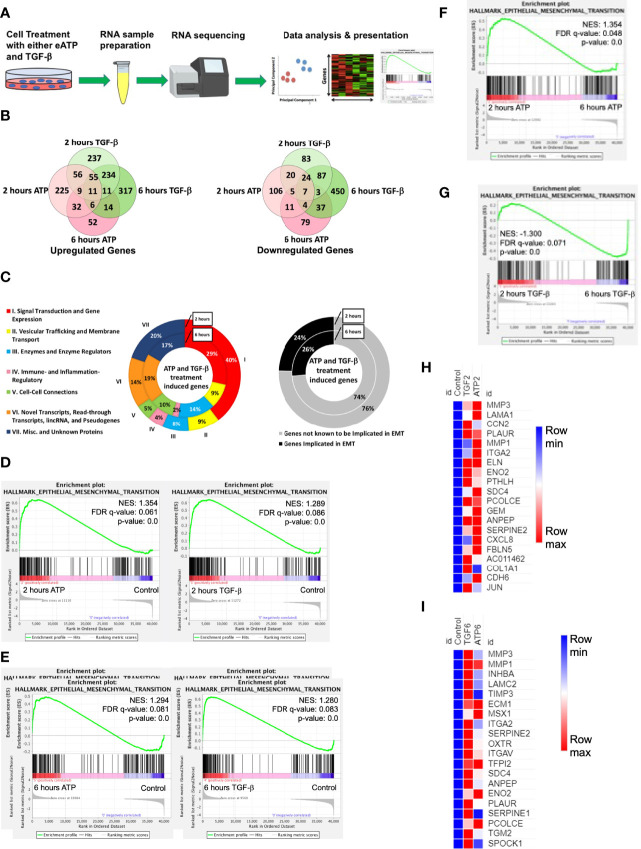
RNAseq study. A549 cells were treated with either 0.5 mM ATP or 10 ng/mL TGF-β for 2 or 6 hours with three replicates per treatment condition. After the treatment, polyA-containing RNA was isolated and then sent to a commercial service company for RNAseq analysis as described in detail in the Materials and Methods. The general RNAseq results are presented in Venn diagrams, pie graphs, GSEA plots, and heatmaps. NES: normalized enrichment score. **(A)** Workflow for RNA-sequencing study.** (B) **Venn Diagram of genes significantly upregulated (left) or downregulated (right) by either ATP or TGF-β at 2 or 6 hours. **(C)** pie graph that shows the 131 EMT related genes upregulated by both eATP and TGF-β at 2 hours and 42 genes at 6 hours (left), and percentage of known EMT genes at both times (right). **(D)** Gene set enrichment analysis (GSEA) plots for the “Hallmark - Epithelial mesenchymal transition” signature, 2 hours ATP compared to control (left) and 2 hours TGF-β compared to control (right). (**E–G): **GSEA plots for the “Hallmark - Epithelial mesenchymal transition” signature. **(E)** 6 hours ATP compared to control (left) and 6 hours TGF-β compared to control (right). **(F)** 2 hours ATP compared to 6 hours ATP. **(G) **2 hours TGF-β compared to 6 hours TGF-β. (**H, I): **Heatmap** **showing top 20 enriched genes from the ”Hallmark - Epithelial mesenchymal transition” signature in **(H)** 2 hours ATP and 2 hours TGF-β and **(I)** 6 hours ATP and 6 hours TGF-β. For heatmaps **(H)** and **(I)**, FPKM values for all three replicates of each gene are shown in the **Supplemental Tables 2**, **3**.

First, we wanted to determine if and how eATP induces changes at the gene expression level as compared with TGF-β. Such a study was never done at the transcriptome level. To that end, RNA sequencing (RNAseq) analysis was performed (**Figure 1A**).

At 2 hours of treatment, TGF-β significantly upregulated a total of 613 genes while eATP upregulated 394 genes (**Figure 1B** left). In addition, among these genes, 131 were common between both treatments at 2 hours (**Figure 1B** left and **1C** left). At 6 hours, TGF-β induced 648 genes whereas eATP induced 135 genes. Between 2 hours and 6 hours, TGF-β significantly induced roughly the same number of genes while eATP significantly induced only about 1/3 of the genes at 6 hours compared with those of 2 hours. At 6 hours, 42 genes were significantly upregulated by both inducers (**Figure 1B** left and **1C** left). These large changes over time in the eATP-induced gene expressions correlate with the invasion and morphology changes induced by eATP (**Figures 3**, **4**).

In comparison, at 2 hours, TGF-β significantly downregulated 229 genes while eATP downregulated 177 genes (**Figure 1B** right). At 6 hours, TGF-β significantly downregulated 612 genes. In contrast, eATP significantly downregulated 146 genes, a small decrease compared with its 2-hour counterpart (**Figure 1B** right).

Similarly, eATP and TGF-β also downregulated numerous genes, primarily epithelial genes (**Figure S1A, B**).

All these data shown above supports the notion that the state induced by eATP, similar to the one induced by TGF-β, is a partial EMT.

In the gene set enrichment analysis (GSEA) plots for the “Hallmark - Epithelial mesenchymal transition” signature, EMT is more enriched at 2 hours (**Figure 1D**) and at 6 hours (**Figure 1E**) for both eATP and TGF-β treatments compared to the untreated controls. Comparisons between 2 and 6 hours of the same treatment show that EMT is more enriched at 2 hours than 6 hours for ATP (**Figure 1F**), while EMT is more enriched for TGF-β at 6 hours than two hours (**Figure 1G**), suggesting that EMT enrichment/progress induced by ATP is earlier/faster than that for TGF-β. From the gene set enrichment analysis, we found the top 20 most enriched genes in common between ATP and TGF-β at both 2 hours and 6 hours (**Figures 1G, H**). The results from the top 20 enriched genes are consistent with ATP appearing to induce EMT earlier than TGF-β. EMT related genes were more enriched by ATP induction at 2 hours (**Figure 1H**) while TGF-β induced genes were more enriched than ATP at 6 hours (**Figure 1I**). Thus, the heatmap results are consistent with the enrichment analyses (**Figures 1F, G**), again confirming that ATP appears to induce EMT enrichment earlier than TGF-β. In addition, enrichment analysis showed that TGF-β had significantly more genes enriched for EMT in comparison to ATP at 6 hours (**Figure S1C**, right), but not at 2 hours (**Figure S1D**, left).

### Eleven Genes Were Upregulated and Conserved in Inductions by Both eATP and TGF-β

Both eATP and TGF-β induced significant upregulation of a common set of 11 genes at both 2 and 6 hours of treatment (**Table 1**, top half). Among these 11 genes, several are well-known to be involved in EMT, such as *Sox8*, *BMP6*, *MMP10*, and others. Several other genes, however, are less well known or not currently known to be associated with EMT. These include *STC1*, *GJB3*, and *BLOC1S6*. Intriguingly, ATP6V1G2-DDX39B, a long non-translated fusion RNA (LncRNA), was also conserved in this group. This readthrough transcript contains an ATPase gene and the *DDX39B*, an RNA splicing gene, raising questions as how this RNA participates in the induction and regulation of EMT.

RNAseq analysis also identified 7 genes that were conserved and significantly downregulated by both eATP and TGF-β at both times (**Table 1**, bottom half). Some of these genes or LncRNAs are also unknown for their functions in EMT.

A closer examination of the RNAseq results revealed that 6 other EMT related genes were also upregulated but not significantly at both times (**Table 2**). One gene is an exception in this category: HGF, which was significantly downregulated by both eATP and TGF-β at 6 hours.

**Table 2:** EMT genes significantly changed by both the ATP and TGF- β treatments (but may not be changed significantly at all time points).

		log2(FC) values			
Gene Symbol	Gene Name	2 hr ATP	6 hr ATP	2 hrTGF-Beta	6 hrTGF-Beta
BMP2	Bone morphogenic protein 2	**1.66**	0.61	**1.51**	**1.66**
HGF	hepatocyte growth factor	0.07	**-1.28**	-0.24	**-2.70**
IL6	interlukin 6	**2.10**	0.60	0.99	**2.08**
MMP1	Matrix Metalloproteinases 1	**3.43**	**3.30**	0.93	**3.72**
MMP3	Matrix Metalloproteinases 3	**2.54**	**2.68**	1.18	**4.36**
MMP10	Matrix Metalloproteinases 10	**1.86**	**1.87**	**1.10**	**2.51**
NFKB1	Nuclear Factor Kappa B Subunit 1	**1.49**	0.05	**1.13**	0.97

Those values shown in bold face are statistically significantly different from the untreated controls.

Another category of EMT related genes were also identified (**Table 3**). These genes were either upregulated or downregulated by TGF-β significantly. In comparison, they were regulated in the same directions by eATP but not significantly (**Table 3**).

**Table 3:** EMT genes significantly changed by TGF- β treatment only (but may not be changed at both time points).

		log2(FC) values			
Gene Symbol	Gene Name	2 hr ATP	6 hr ATP	2 hr TGF-Beta	6 hr TGF-Beta
NOTCH3	Notch 3	-0.03	-0.24	-0.15	**-1.42**
PDGFA	Platelet-derived growth factor A	-0.13	0.49	**1. 03**	**1.28**
PDGFB	Platelet-derived growth factor B	0.44	0.00	**3.59**	**4.06**
SERPINE1	serpin family E member 1	0.27	0.44	**2.48**	**3.50**
SHH	Sonic Hedgehog	-0.29	-0.76	**1.51**	0.38
SMAD7	SMAD family member 7	-0.82	-0.13	**1. 11**	**1.58**
SNAI1	Snail	0.21	0.50	**2.85**	**3.13**
SNAI2	Slug	0.58	0.58	**2.62**	**2.97**
TNF	Tumor necrosis factor	0.47	0.30	**1.46**	**1.29**
VEGFA	Vascular endothelial growth factor A	0.02	0.32	**1.29**	**2.39**
VEGFC	Vascular endothelial growth factor C	0.47	0.23	-0.52	**-1.08**
VIM	Vimentin	-0.10	0.18	0.36	**1. 06**
WNT3	Wnt family member 3	0.25	-0.40	**1. 11**	0.63
WNT5A	Wnt Family Member 5A	-0.38	-0.17	-0.71	**-1.11**
WNT7A	Wnt family member 7A	-0.55	**1.51**	**2.64**	**4.78**
WNT9A	Wnt family member 9A	0.59	0.92	**1.87**	**1.79**

Those values shown in bold face are statistically significantly different from the untreated controls.

Four known epithelial genes were also identified to be downregulated by both eATP and TGF-β, but not to a similar degree (**Supplemental Table 1**)

All these RNAseq results indicate that eATP induces and regulates EMT at the gene expression level similarly but also differently to TGF-β.

### Metabolomics Analyses of eATP Treated A549 Cells Show Changes Associated With EMT

**Figure 2:**
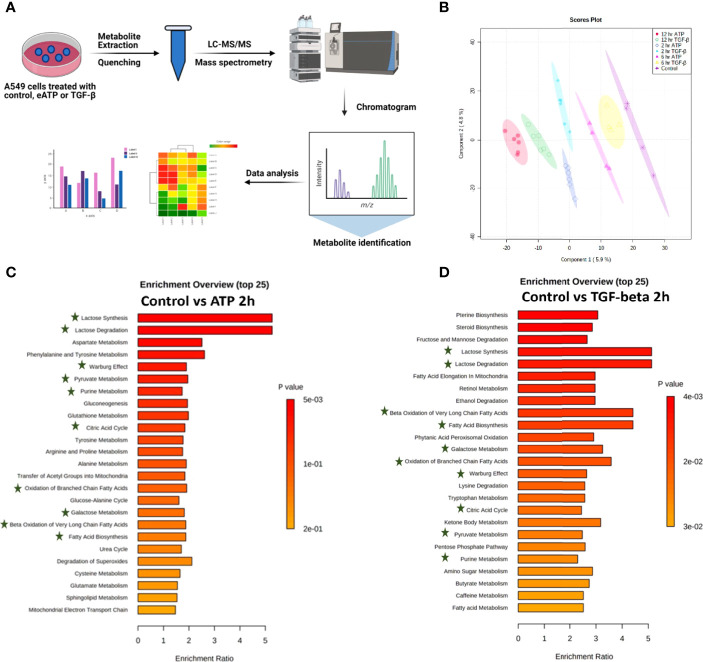
Metabolomics analysis of metabolic changes induced by Eatp. A549 cells were treated with either ATP or TGF-β for 0 (control), 2, 6 and 12 hours. Specially prepared cell lysates were subjected to a metabolomics analysis as described in Materials and Methods. The metabolomics data were presented in different ways to show their EMT-related features, similarities between ATP and TGF-β, and differences from the control. **(A)** Schematic presentation of the design of the metabolomics study. **(B)** PLS-DA plot for eATP or TGF-β treated samples at different treatment times as compared to the untreated control. **(C, D)** Metabolic pathway enrichment analysis of ATP treatment at 2 hr **(C)**, and enrichment by TGF-β at 2 hr **(D)**. Shared metabolic pathways enriched in both conditions are highlighted with asterisks.

Metabolic profiles of eATP- or TGF-β-treated cancer cells induced at various times are “snapshots” of cancer cells’ total sum of metabolic changes and reflections of metabolic and phenotypic changes during EMT induction at those time points. For these reasons, the metabolomics study was conducted (**Figure 2A**). Partial least-square discriminant analysis (PLS-DA) was performed on the data collected by performing negative ion mode LC-MS/MS. The PLS-DA analysis showed that control treated A549 cancer cells could be separated (based on their metabolite abundances) from ATP- and TGF-β-treated cells after 2, 6 and 12 hours of treatment (**Figure 2B**). Similarly, PLS-DA analysis was performed on data collected by positive ion mode (**Figure S2**)

Metabolic pathway enrichment analyses show that, among the 25 most enriched pathways in each treatment, about half of those pathways are either identical or similar between control vs 2hr ATP and control vs 2hr TGF- β treated A549 cells (**Figures 2C, D**). Those identical metabolic pathways are concentrated in the glucose metabolism area, including glycolysis/gluconeogenesis, TCA cycle, glutamine, pyruvate, and purine. TGF-β induces these for EMT (11, 12). These pathways are known to be inducers of EMT and thus this shows similarity in EMT-like alterations induced by ATP and TGF- β in A549 cell metabolism.

### eATP Induced Similar Cell Invasion to TGF-β in Two Human Lung Cancer Cell Lines Tested

**Figure 3:**
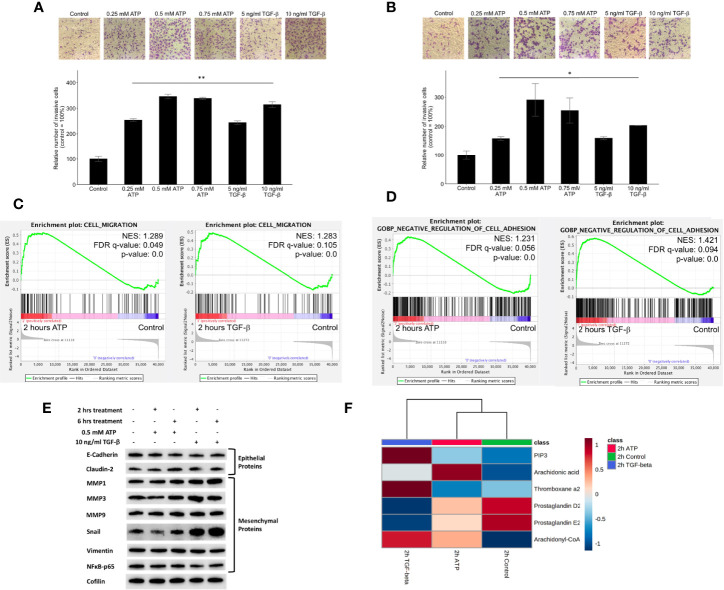
Extracellular ATP induces dose-dependent invasion faster than TGF-β. Human NSCLC A549 and H1299 cells were grown in Transwells with collagen Matrigel coated membranes for 20 hours (invasion assay) in the presence or absence of ATP or TGF-β at various concentrations. After the treatment, invaded cells were fixed, stained, and visually counted and averaged. RNAseq analysis was used to identify corresponding gene expression changes. GSEA plots for the “cell migration” and “negative regulation of cell adhesion” signatures were performed to match/verify the observed phenotypic changes. NES: normalized enrichment score. *P < 0.05, **P < 0.01. **(A, B)** eATP and TGF-β induce cell invasion in A549 cells **(A)** and in H1299 cells **(B, C)** GSEA plots, 2 hours ATP compared to control (left) and 2 hours TGF-β compared to control (right). **(D)** GSEA plots for the “Negative regulation of cell adhesion” signature, 2 hours ATP compared to control (top) and 2 hours TGF-β compared to control (bottom). **(E)** Western blot analysis of selected proteins related to EMT. Cofilin served as a loading control. The quantification of these proteins can be found in the **Supplemental Figure S3**. **(F)** Metabolomics heatmap for 6 metabolites involved in cell migration in differently treated cells.

Transwell assays showed that the invasion rates were eATP dose-dependent in both A549 cells (**Figure S3A**) and H1299 cells (**Figure S3B**). Transwell invasion assays show that eATP induced dose-dependent invasions similar to TGF-β in NSCLC cell lines A549 (**Figure 3A**) and H1299 cells (**Figure 3B**). Gene set enrichment analysis (GSEA) reveals that genes involved in cell migration (**Figure 3C**) and in negative cell adhesion regulation (**Figure 3D**) were significantly enriched by eATP and TGF-β, supporting the increased invasion, compared with the untreated controls, induced by both molecules is likely through similar mechanisms.

Western blot analysis indicated that selected E-type proteins were in general downregulated while M-type proteins were upregulated by eATP, and eATP induced changes are in general in the same direction as TGF-β (**Figure 3E**) and its quantification of (**Figure S3C**), further supporting the role of eATP in EMT induction.

Finally, the increased invasion was confirmed by the metabolomics analysis of invasion/migration-related metabolites, whose abundances were altered in a similar way in both 2hr ATP- and 2hr TGF-β induced samples (**Figure 3F**). These results also indicate that invasion as an EMT-required phenotypes could be induced by eATP in the range of eATP concentrations found in TME (15–18).

### eATP Induced Formation of Filopodia-Like Protrusions Earlier Than TGF-β in Two Human Lung Cancer Cell Lines

**Figure 4:**
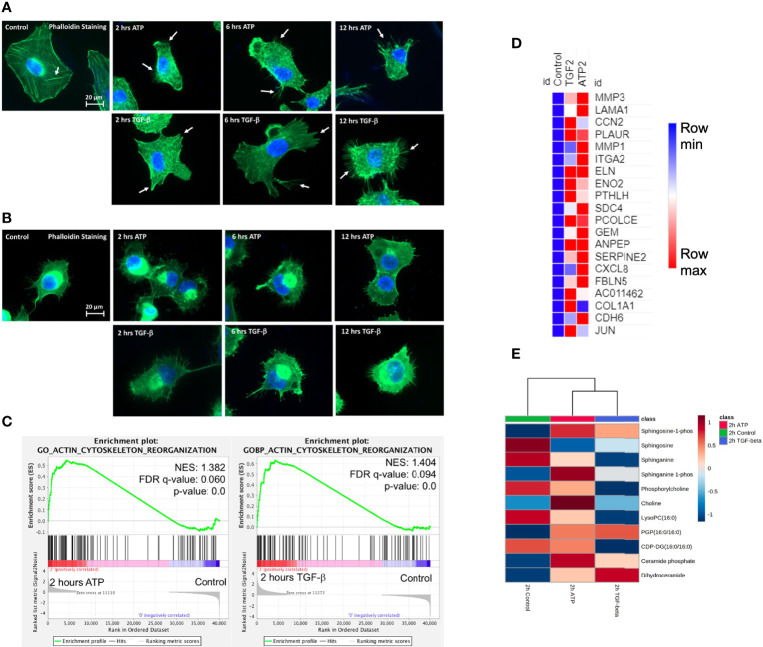
Extracellular ATP induces time-dependent formation of filopodia similar to TGF-β confirmed by transcriptomics and metabolomics analyses. Human NSCLC A549 and H1299 cells were grown on coverslips in cell culture plates were treated with either eATP or TGF-β for various hours. After treatment, fluorescence microscopy was used to visualize time-dependent formation of filopodia-like protrusions in eATP- or TGF-β-treated A549 and H1299 cells. NES: normalized enrichment score. **(A, B)** Fluorescence microscopy of time-dependent formation of filopodia-like protrusion formation in eATP-treated A549 cells **(A)** and H1299 cells (**B**). Untreated and TGF-β treated cells served as negative and positive controls. Arrows point to distinctive protrusions of the cells. The quantification of the filopodia formation can be found in the **Supplemental Figure S4**. **(C)** Transcriptomics GSEA plots for the “GO Actin Cytoskeleton Reorganization” signature derived from RNAseq data, 2 hours ATP compared to control (top) and 2 hours TGF-β compared to control (bottom). **(D)** Heat mal of the top 20 enriched genes in common between ATP and TGF-β for actin cytoskeleton reorganization. The FPKM values of three replicates of each gene are shown in **Supplemental Table 4**. **(E)** Heatmap of metabolomics analysis showing metabolites involved in actin cytoskeletal rearrangement altered by ATP and TGF-β in A549 cancer cells after 2 hours of treatment.

Filopodia are “feet-like” cell structures necessary for loss of cell polarity and cytoskeleton remodeling, and cell migration and invasion and are a cellular morphology feature induced in EMT. We previously demonstrated eATP-induced formation of filopodia-like protrusions in A549 cells (29). But we did not know when the protrusions were induced or if the timing of protrusion formation induced by eATP or TGF-β differ from each other. The time course study revealed that the protrusions were formed as early as 2 hours in A549 cells treated with eATP and these protrusions persist for at least 12 hours (**Figure 4A**). Similar changes were also observed in H1299 cells (**Figure 4B**) with the difference of some protrusions being formed even without eATP or TGF-β treatment. The pre-existing protrusions in H1299 cells were previously documented (48). TGF-β concentrations used in these assays were most commonly reported in the literature. This assay provides a piece of visual evidence for the first time that eATP induces time-dependent EMT-related and EMT-required cellular morphology changes, and the induced filopodia formation is similar to those induced by TGF-β in timing and degree (**Figure S1H**).

RNAseq GSEA analysis shows that actin-cytoskeleton reorganization related genes are enriched 2 hours after ATP or TGF-β treatment (**Figure 4C**). The top 20 enriched genes in common between ATP and TGF-β for actin cytoskeleton reorganization show similar changes (**Figure 4D**). Metabolomics analysis on cell actin-cytoskeleton rearrangement showed that, among 12 metabolites in this group, 11 metabolites show concentration changes induced by eATP, either increased or decreased, in the same direction compared with TGF-β induction relative to the control at 2 hours (**Figure 4E**). These metabolites are known to be changed in EMT. Thus, these results indicate that actin-cytoskeleton rearrangement has occurred in these cells treated with either molecule as a result of EMT.

### ATP and TGF-βShow Additive Effects on Cell Invasion at Lower Concentrations but Not at Higher Concentrations

**Figure 5:**
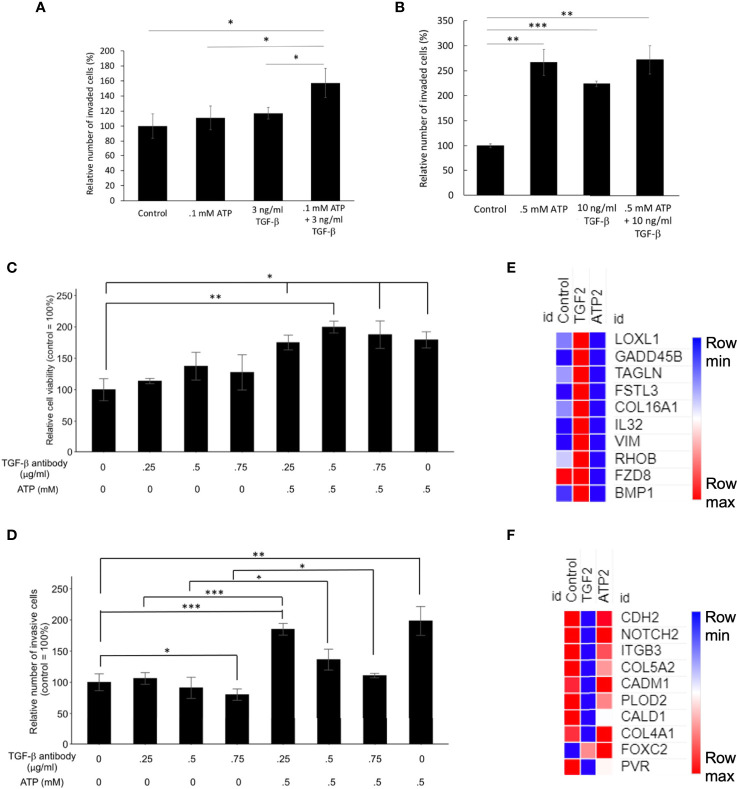
Mechanism study I: Similarities and differences in EMT induced by eATP or TGF-β. Human NSCLC A549 and/or H1299 cells grown in either regular cell culture plates or transwell plates were treated with either ATP, TGF-β, or both at various concentrations and measured for their respective intracellular ATP levels or additive effect in the invasion assay. In a different study, A549 cells were treated with anti-TGF-β neutralizing antibody at various concentrations in the presence or absence of different concentrations of eATP. 24 hours after the treatment, treated cells were assayed for their intracellular ATP levels or their cell viability by resazurin assay. *P < 0.05, **P < 0.01. ***P <.001. **(A, B)** eATP and TGF-β show an additive effect in inducing invasion at lower concentrations **(A)** while eATP and TGF-β do not show an additive effect in inducing invasion at higher concentrations **(B)**. **(C, D)** Extracellular ATP increased cell viability of cells treated with TGF-β neutralizing antibody **(C)**. Extracellular ATP restored the invasiveness of A549 cells treated with TGF-β antibody **(D)** in A549 cells. **(E, F)** Heatmaps of EMT-related genes upregulated by TGF-β at 2 hours **(E)**, and EMT-related genes upregulated by ATP at 2 hours **(F)**. The FPKM values of three replicates of each gene are shown in **Supplemental Tables 5**, **6**, respectively.

At concentrations significantly lower than their respective optimal concentrations for EMT induction, the combined treatment of eATP and TGF- β produced more invasion than the individual treatment alone (**Figure 5A**). However, this additive effect disappeared when regular concentrations were used (**Figure 5B**), suggesting that eATP and TGF-β are very likely to act by using, at least in part, the same pathways for inducing invasion. These same pathways include eATP activated purinergic receptor signaling (13–15). Both eATP and TGF-β are known to use purinergic receptor signaling for their EMT induction (12–14). The concentrations of eATP and TGF-β used in this study were selected for the following reasons. Intratumoral eATP concentrations have been reported to be in the range of 0.2 – 0.6 mM (16–19). Thus, 0.5 mM is the eATP concentration found in real tumors. 0.5 mM has been the eATP concentration we used in our previous studies (25–29). This concentration has been chosen by us and accepted by the field. These concentrations are also consistent with the concentrations used in the RNAseq and Metabolomics analysis in this study.

### eATP Restored Invasiveness of A549 Cells Reduced by Antibodies Against TGF-β

To further characterize the functional relationship between eATP and TGF-β in invasion, a cell viability assay was conducted. This assay result revealed that normal cell viability/proliferation was not stopped in A549 cells by the addition of TGF-β neutralizing antibodies, but the cell viability was further increased by the addition of eATP (**Figure 5C**). In comparison, cell invasiveness was reduced by TGF-β neutralizing antibody treatment but the reduced invasiveness was partially restored by the addition of ATP (**Figure 5D**). However, this restoration was reduced by the increasing amount of the antibodies. These results indicate that eATP could rescue A549 cells, at least in partial, from the invasion-inhibitory activities exerted by TGF-β neutralizing antibodies. This also strongly suggests that eATP’s invasion inducing activity is partially dependent upon TGF-β signaling. These results are consistent with those found in invasion studies (**Figure 3**), cell morphology studies (**Figure 4**), and the additive effect study (**Figures 5A, B**).

RNAseq analysis revealed that, after 2 hours of treatment, a set of genes involved in EMT were enriched by TGF-β but not by eATP, while another set of EMT genes were enriched by eATP but not by TGF-β (**Figure 5G**). These results, in combination with **Figures 1G, H**, show that ATP and TGF-β use some identical and some different genes to induce EMT, providing an explanation for the additive effect observed at low doses and ATP’s ability to rescue invasion when TGF-β signaling is not present.

### Extracellular ATP Induces Dose-Dependent Elevation of Intracellular ATP Concentrations While TGF-β Does Not

**Figure 6:**
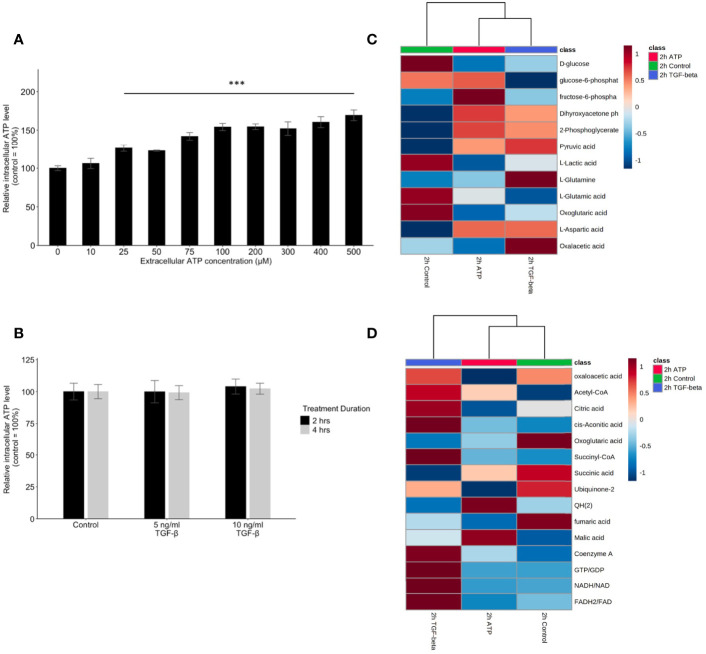
Mechanism study II: similarity and difference between eATP and TGF-β in intracellular ATP and in ATP synthesis-related metabolites. A549 cells were treated with either various concentrations of ATP or TGF-β. 24 hours after the treatment, cells were lysed and measured for their intracellular ATP concentrations. A549 cells were also treated by ATP and TGF-b for 2 hours and then their specific metabolites analyzed. ***P <.001. **(A, B)** Intracellular ATP levels of cells treated by various concentrations of ATP **(A)** and TGF-β **(B)**. **(C, D)** Heatmaps of concentrations of metabolites involved in glycolysis **(C)** or TCA cycle **(D)** in cells treated with ATP or TGF-β for 2 hours compared with the untreated control.

To compare the similarities and differences between eATP and TGF-β in their respective mechanisms in EMT induction, the treatment followed by intracellular ATP (iATP) measurements were performed. The assay showed that eATP induced dose-dependent elevation of iATP concentrations in A549 cells (**Figure 6A**) while TGF-β treatment did not change iATP level (**Figure 6B**). The same result was observed in H1299 cells (**Figures S4A, B**). These results suggest that, although eATP induces EMT by possibly using similar mechanisms used by TGF-β, eATP actions are aided by increased iATP concentration. In contrast, TGF-β’s EMT-inducing mechanisms do not involve iATP level elevation. Therefore, the differences in iATP levels may account for the differences observed in the invasion and filopodia formation induced by the two molecules (**Figures 3**, **4**), since iATP can function as either an energy source for cell movement, a phosphate donor, a signal amplifier in signal transduction, and even a transcription cofactor.

### eATP Induced Transcriptomics and Metabolomics Changes in ROS Pathways and Corresponding Metabolites

Glycolysis, glutaminolysis, and TCA cycle are known to be altered during EMT (11). Most of the metabolites in glycolysis and glutaminolysis changed their concentrations in the same direction 2 hours after the treatment by either ATP or TGF-β (**Figure 6C**). Larger differences can be found in the TCA cycle metabolism (**Figure 6D**). This may be due to the fact that one key function of the TCA cycle is for mitochondrial ATP synthesis but this synthesis is largely unnecessary for the eATP-treated cells because of the macropinocytosis mediated eATP internalization. Thus, it is not surprising that control and eATP treated samples were clustered together much more than TGF-β-treated samples.

**Figure 7:**
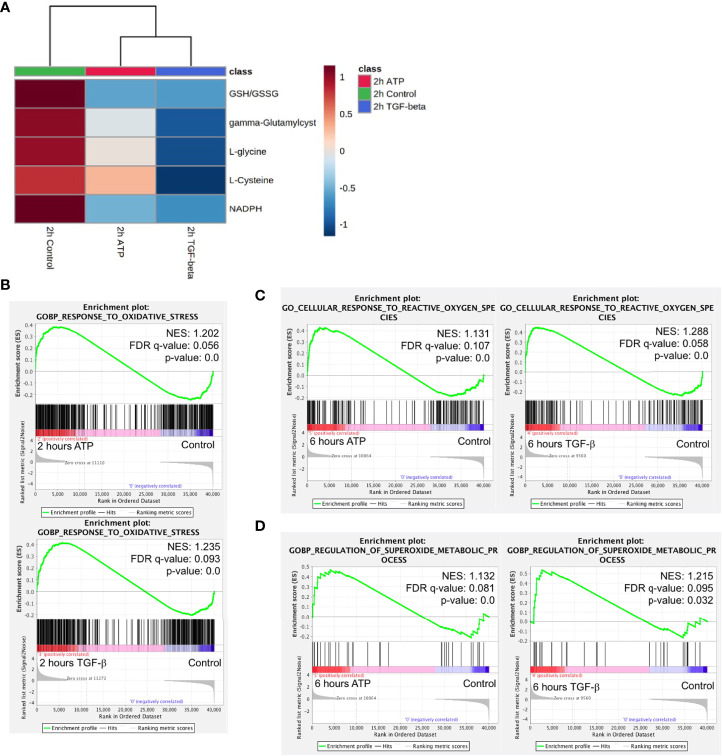
Mechanism study III: similarity and difference between eATP and TGF-β in ROS-related metabolites and genes. A549 cells were treated with either ATP or TGF-β for 2 hours. Then analyzed for their respective transcriptome and metabolome profiles. **(A)** Heatmap of major metabolites involved in the ROS pathway and GSH/GSSG ratio (a marker for redox homeostasis in cancer cells). **(B–D)** GSEA of RNAseq data for the enrichment of oxidative stress **(B)**, cellular response to ROS **(C)**, and superoxide metabolism **(D)**.

ROS is well known to be involved in EMT (11). Metabolomics analysis reveals that concentrations of major metabolites in the ROS pathway changed to the same direction in both ATP- and TGF-β-treated samples after 2 hours of treatment (**Figure 7A**), indicating an increasing ROS status.

GSEA of RNAseq data also show that response to oxidative stress, cellular response to ROS, and superoxide metabolic processes are enriched (**Figures 7B–D**), matching and supporting the metabolic finding.

This indicates that, regarding the ROS and oxidative stress status, eATP-induced and TGF-β-induced cells were in very similar metabolic state, presumably an EMT state.

**Figure 8:**
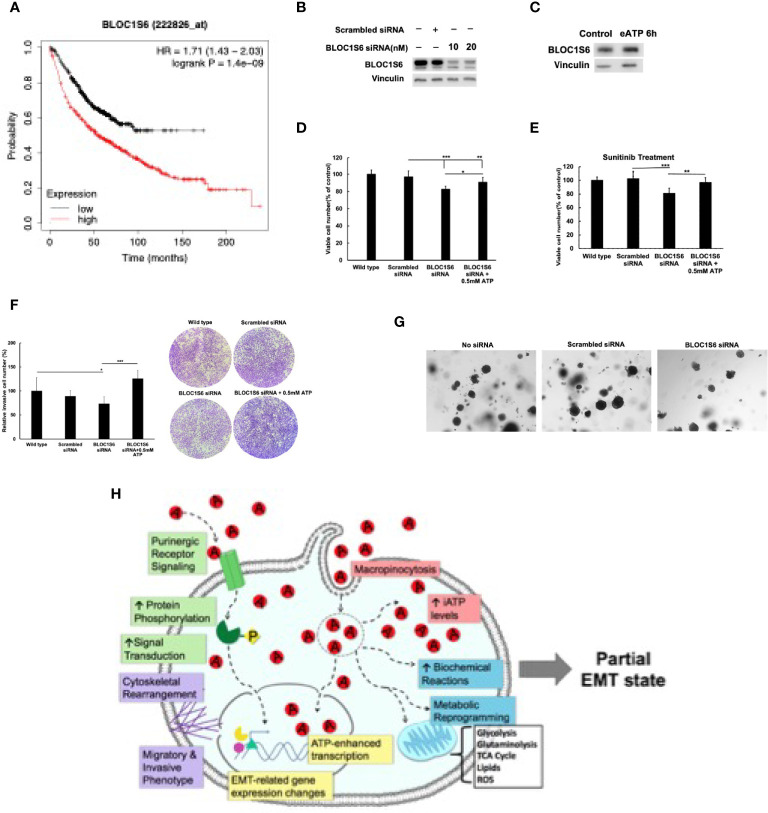
*BLOC1S6* gene is involved in the eATP induced proliferation, drug resistance invasion and colony formation in A549 cells. *BLOC1S6* gene was knocked down or overexpressed in A549 cells. BLOC1S6 protein levels in these cells were measured by western blots. The KD A549 cells were tested in various assays to assess its potential roles in EMT and CSC formation compared with regular A549 cells. **(A)** The inverse proportional relationship between BLOC1S6 mRNA level and survival of lung cancer patients. Kaplan-Meier plots online tool (https://kmplot.com/analysis/) was used to analyze the relationship between Bloc1s6 genes and the overall survival rate of lung cancer. **(B)** Protein expression levels of BLOC1S6 under different siRNA treatment conditions. **(C)** Protein expression levels of BLOC1S6 with or without 0.5mM ATP treatment. **(D)** Cell viability/proliferation assay of KD cells with or without eATP treatment. **(E)** Cell viability/proliferation assay of KD cells treated with anticancer drug Sunitinib at 20 mM in the presence or absence of eATP. *P < 0.05, **P < 0.01,***P <.001. **(F)** Invasion assay of KD cells with or without eATP treatment. **(G)** Soft agar anchor-independent colony assay for testing KD cells’ colony formation capability in KD and non-KD cells. Quantification of the assay can be found in **Supplemental Figure S6**. **(H)** A hypothetical model for the mechanisms used by eATP to induce EMT. Detailed explanation for the model can be found in Discussion.

As a first step for assessing the functions of BLOC1S6 in EMT and CSC, we knocked down *BLOC1S6* gene (**Figure 8B**). *BLOC1S6* is a gene with its functions basically unknown but its expression levels found to be inversely proportional in lung cancer patients’ survival (**Figure 8A**) (45). We also used eATP to treat A549 cells and found that the treatment led to increased BLOC1S6 protein (**Figure 8C**), confirming the validity of our RNAseq data (**Table 1**). The KD of *BLOC1S6* also resulted in reduced cell proliferation (**Figure 8D**), Drug resistance to FDA approved target drug sunitinib (**Figure 8E**) (27), invasion (**Figure 8F**), and the capability of forming colonies (**Figure 8G**). All these strongly suggest that BLOC1S6 is involved in and contributes to EMT and CSC formations.

Based on all these new findings and our previous findings, we propose a new hypothetical model to explain the mechanisms used by eATP to induce EMT (**Figure 8H**).

## Discussion

In our previous study, we showed that eATP induced all the *in vitro* key features of EMT (29) as defined by the consensus of the field (3). These key features include cell detachment, loss of apical-basal polarity and cytoskeleton remodeling, cell migration and invasion, upregulation of some M protein markers and reduction of some E markers (29). However, we did not show these changes at gene expression and metabolic levels. In this study, we continue and further expand the eATP study into transcriptomics and metabolomics, and to establish eATP as an inducer and regulator of EMT on a firmer basis.

As described in the Introduction, several unique features of eATP and macropinocytosed eATP support them as being a potential inducer and regulator of EMT. First, ATP is an extracellular messenger, secreted downstream of either a TGF-β-dependent pathway (13, 14), or independent pathways (15), for activating purinergic receptor (PR) mediated signaling, which has been implicated in EMT (49). The PR signaling provides a major part of action specificity for ATP-triggered signaling and actions. ATP is also a versatile transcription cofactor, participating in a wide variety of transcriptional activities such as DNA unwinding, transcription initiation, elongation, and termination (30–34), providing a second mechanism for action specificity of ATP. Third, ATP is a protein phosphorylation donor involved in a large portion of intracellular signaling pathways. This way, in PR signaling, eATP not only triggers PR activation, but also enhances PR signaling by increasing phosphorylation of proteins/enzymes involved in PR signaling when eATP is internalized by macropinocytosis to elevate intracellular ATP concentrations. We previously demonstrated this activity in other cell growth signaling pathways (27). Furthermore, ATP is also a cofactor in energy-required enzymatic reactions, accelerating reaction rates of those ATP requiring enzymatic reactions. These properties make ATP stand out as a candidate for functioning as an inducer and regulator for EMT, a process that relies on well-regulated changes in signal transduction, gene expression (transcription), translation, and metabolism. More directly related to cell motility and metastasis, ATP is a well-known danger signal for bacteria and cancer cells (46, 47). Elevated ATP concentrations in the environment functions as a warning signal to bacteria and animal cells for the incoming danger and informing them to flee for a safer environment. It can be envisioned that this conserved activity is hijacked and utilized by cancer cells to signal imminent danger of their original sites within tumors when the conditions is deteriorating due to hypoxia and shortage of nutritional supply. These deteriorations results in cell lysis and ATP release, sending departure signals leading to invasion and metastasis. Some major remaining questions we tried to address include: how much, at what time and levels, and in what relationship to TGF-β, eATP contributes to EMT? Our current study was one of the first steps towards answering these key questions for better understanding of the eATP-mediated EMT induction process by using a combinatorial study strategy of RNAseq, metabolomics, and functional assays.

The RNAseq analysis show that, like TGF-β, eATP upregulated and downregulated many genes. Most of these genes are regulated similarly by eATP and TGF-β (**Tables 1**–**3**), strongly suggesting that eATP induced and regulated the same process as TGF-β, namely early phase of EMT at 2 and 6 hours. In addition, like TGF-β, eATP exhibited time-dependent gene expressions. The 6-hour treatment led to significantly altered expression of some new genes that did not show up at the 2-hour treatment and downregulation of some other genes. These expression pattern changes suggest that eATP not only induces the onset of EMT, but also temporally regulates EMT, orchestrating the progress by expressing the right genes at the right times and at right levels, just like TGF-β. It is interesting to find out that the percentage of shared upregulated EMT genes by eATP and TGF-β was about 24% or 26% (**Figure 1C** right), or 23% for the downregulated EMT genes (**Figure S1B**). These values are very similar to those reported by others with large numbers of single cell samples and time courses: 22% (50).

On the first look, eATP induced fewer significant changes in upregulated and downregulated genes compared with TGF-β. A closer examination of the RNAseq data reveals that eATP induced changes of expression in about as many genes as TGF-β, but just not to the level of statistical significance determined by the RNAseq software (unpublished observations). Similar phenomenon was also observed in metabolomics data (**Figure 4E** and **5D**). The multi-functionality and multi-locality of ATP and macropinocytosis-internalized eATP might be behind these differences. It is possible that the elevated iATP concentrations (**Figure 5D**) lead to more protein phosphorylation in signal transduction and accelerated biochemical reactions while maintaining the expression of the enzyme genes and metabolites involved in these reactions at levels lower than those found in TGF-β-treated cells (**Table 1**, and **Figure 4E** and **5D**), as these changes make higher gene expression levels unnecessary.

In addition, for some of those M-type genes significantly altered by both eATP and TGF-β, eATP-induced genes tend to show higher Log2fc values at 2 hr than those induced by TGF-β, while genes induced by TGF-β tend to have higher Log2fc values at 6 hr than eATP-induced genes (**Table 1**). This pattern of gene expression suggests that eATP induces gene expression changes earlier and possibly faster than TGF-β, consistent with the observation that eATP induced faster morphological changes than TGF-β [**Figure 3** and (29)]. This phenomenon may also be due to the same multi-locational and multi-functional property of ATP.

Unlike the RNAseq profiles at 2 and 6 hours after the inductions, which reflected the changes in early and later stages of gene expression during EMT, the metabolomics profile represents changes at the metabolic and therefore phenotypic levels associated with EMT (48, 49). The metabolomics data provides evidence, in addition to the RNAseq gene expression data, that eATP induced a metabolic profile similar to that induced by TGF-β (**Figure 2**). The specific altered pathways and metabolite levels, compared with TGF-β, are clear indications that the metabolic state induced by eATP is similar to that induced by TGF-β, and is indeed a state corresponding to EMT. These findings have never been reported before.

We recently reported the observation that eATP induced both migration and invasion (29), two key features of EMT. Our current invasion assays further expand the study by showing dose-dependent comparison between eATP and TGF-β in not only A549 cells, but also in a second lung cancer cell line H1299 (**Figure 3A, B**). The new result shows that this is a multi-cell line and a potentially multi-cancer type phenomenon. The doses of eATP used in this study were the same as the concentration range of eATP found in TME (16–19), implying its roles in real tumors. Our subsequent fluorescent microscopy study revealed that the eATP treatment led to an earlier formation of filopodia-like protrusions, in A549 and H1299 cells in a time-dependent manner (**Figure 4A, B**) than TGF-β. This result provides a first piece of visual evidence for early phase EMT-related morphological changes, loss of apical-basal polarity and cytoskeleton remodeling, induced by eATP.

Eleven genes, which were significantly upregulated and completely conserved in eATP and TGF-β treated cells at both 2 and 6 hours, were identified (**Table 1**). Several genes in this group, including *Sox8*, *BMP6*, *MMP10*, and *IL-1A*, are known to play roles in EMT. Other genes such as *BLOC1S6*, are not known to be involved in EMT. It is particularly interesting to find that *ATP6V1G2-DDX39B*, a long noncoding transcript (LncRNA), containing untranslated ATPase and RNA splicing genes, is also included in this group eATP and TGF-β share some functional similarities and differences to each other (**Figure 5**). First, both eATP and TGF-β promote invasion. They enhance invasion induced by the other molecule when added together at low concentrations (**Figure 5A**), but not at higher concentrations. (**Figure 5B**), suggesting potential overlap of the invasion-inducing activities of the two molecules. When TGF-β neutralizing antibodies were added, cell viability/proliferation increases (**Figure 5C**), indicating that TGF-β itself is cell proliferation-inhibitory under the experimental conditions in A549 cells, different from eATP. In addition, the antibodies reduce invasion induced by TGF-β or by eATP (**Figure 5D**). The former activity indicates that TGF-β is invasion-promoting, the same as eATP. The latter activity indicates TGF-β signaling is important for invasion, and the invasion induced eATP also partially relies on TGF-β signaling, although no TGF-β needed to be added to the system.

The faster EMT induction by eATP than TGF-β may be related to macropinocytosis-mediated ATP internalization, which results in a large elevation of intracellular ATP (iATP) levels. The ATP assay confirmed this speculation in that eATP induced large dose-dependent iATP elevations in both A549 and H1299 cells, while TGF-β did not (**Figure 6A, B**). It is conceivable that the highly elevated iATP enhanced protein phosphorylation in signal transduction, accelerated biochemical reactions and cell morphology changes, and increased cell motility. We previously demonstrated that some of these activities were blocked when macropinocytosis, a primary hallmark of cancer metabolism (51), was inhibited (29). A549 and H1299 cells are known to exhibit macropinocytosis (52, 53). In addition, iATP directly participates in induction and regulation of gene expressions in cancer cells as a transcriptional cofactor. All these combined together may account for the greater invasion rates compared with TGF-β (**Figure 3A, B**).

Our KD study with *BLOC1S6* indicates that this gene is very likely to be involved in EMT and CSCs, validating our RNAseq study and our selection of the gene as a candidate for EMT/CSC study. Very large differences between BLOC1S6 expression levels and lung patient survival times (**Figure 8A**) (45) also implies its importance in tumorigenesis. Much more detailed studies such as KO studies *in vitro* and *in vivo* are needed for elucidating the working mechanism(s) of the gene in EMT and CSCs. Nevertheless, this study provides initial evidence and justifies for further investigation.

Based on all the previous studies related to ATP-induced EMT (13, 29) and this study, here we propose a significantly updated hypothetical model for how eATP induces and regulates EMT spatially and temporally in human lung cancer cells (**Figure 8G**). First, eATP, at the concentration range found in TME (16–19), functions extracellularly by binding and activating various purinergic receptors (PR) located on plasma membrane of A549 cells (54–56), leading to PR-mediated specific signaling for EMT induction (49, 57, 58). Exactly which PR(s) are activated depend on the specific eATP concentration, as different PRs have different affinities for ATP. This part of eATP activity is similar or identical to the mechanism of TGF-β mediated PR signaling as TGF-β induces ATP exocytosis and subsequent ATP-PR binding/activating (12, 13) with the exception that eATP levels in the TME may be higher than those achieved by TGF-β -mediated ATP exocytosis, as our recently reported data implies (29). This is because eATP in the TME is from multiple sources (58, 59) in addition to TGF-β-induced exocytosis (12, 13). Simultaneously with the PR signaling, eATP is also internalized by macropinocytosis (19–21, 24–26, 29), greatly enhancing the level of intracellular ATP (iATP) by at least 30-50% within 2-3 hours (25–27, 29). The elevated iATP, in turn, accelerates all biochemical/enzymatic reactions inside the cell partly driven by ATP, including both ATP hydrolysis in metabolic reactions and protein phosphorylation in signal transduction (26, 27, 29). Furthermore, ATP is a versatile transcriptional cofactor, directly participating in and augmenting gene expression by ways of double strand DNA unwinding, transcription initiation, elongation, and other steps in transcription (30–34). All these processes working concurrently at different subcellular locations and at various levels of biological reactions result in induction and spatial and temporary regulation of EMT. While the specificity of the gene expression induced by eATP is likely to be originated from the PR signaling, the intensity of the gene expression is likely to be regulated by the other intracellular functions of eATP and potential negative feedback loops between gene transcription rates and enhanced enzymatic activities induced by augmented protein phosphorylation and/or faster enzymatic reactions (and therefore altered metabolite levels) driven by higher iATP levels. Thus, this model not only explains how eATP induces TGF-β-like EMT, but also explains why eATP induces EMT somewhat differently, due to both extracellular and intracellular actions of ATP, from TGF-β-induced EMT at the levels of transcription and biochemical reactions, resulting in earlier morphological/functional changes. Additional studies are needed for the final validation of this hypothetical model.

## Conclusions

eATP is abundantly present in the TME, is an evolutionarily conserved and selected but previously under-recognized molecule that has been emerging as a powerful inducer and regulator of EMT. Its roles in EMT induction are “amplified” by its internalization through macropinocytosis and subsequent drastic elevation of intracellular ATP levels. Results of this study not only demonstrate the multi-functional and multi-locational nature of eATP, but also exhibit the flexibility of cancer cells, which are able to use either TGF-β or eATP or both, whichever is present in the TME at appropriate concentrations or in various ratios to induce the onset of EMT. The RNAseq and metabolomics analyses not only reveal the eATP induces EMT from expression of specific genes to representative metabolite changes similar to those induced by TGF-β. The functional assays further validate the results from transcriptomics and metabolomics. New findings documented in this study will compel us to rethink exactly how EMT is induced and regulated in tumors, and how it will enable us to develop novel and effective anticancer and anti-metastasis strategies by targeting eATP (60) or its induced genes.

## Data Availability Statement

The original contributions presented in the study are publicly available. The RNAseq data is stored at NCBI GEO (Gene Expression Omnibus) with accession number: GSE160671.

## Author Contributions

ME: Design and execution of most experiments related to functional assays and data analysis of RNAseq, figure generation, manuscript writing and editing. JS: Performance of BLOC1S6 study, some supplementary assays, RNAseq related experiments and uploading RNAseq data to GEO database, writing and submission of manuscript. HSG: RNAseq enrichment analysis. XC: Conceptualization and Design of the study, supervision and coordination of the study, participation in writing and submission of the manuscript, and major funding support of the study. All authors contributed to the article and approved the submitted version.

## Funding

For ME, John J. Kopchick Award, Ohio University Student Enhancement Award, Ohio University Provost’s Undergraduate Award, Ohio University Honors Tutorial College Dean’s Research Fund. For JS, Ohio University Original Work Grant, and Graduate Student Research Grant. For PS, John J. Kopchick Award, Ohio University Student Enhancement Award. For XC, NIH grant R15 CA242177-01.

## Conflict of Interest

The authors declare that the research was conducted in the absence of any commercial or financial relationships that could be construed as a potential conflict of interest.

## Publisher’s Note

All claims expressed in this article are solely those of the authors and do not necessarily represent those of their affiliated organizations, or those of the publisher, the editors and the reviewers. Any product that may be evaluated in this article, or claim that may be made by its manufacturer, is not guaranteed or endorsed by the publisher.

## Supplementary Material

Supplementary Figure 1 GSEA plots of gene enrichment in cells treated with either eATP or TGF-β. GSEA plots were made the same way as and . **(A)** Downregulated genes. **(B)** Downregulated genes involved in or not known to be involved in EMT. **(C)** Gene enrichment at 2 hours. **(D)** Gene enrichment at 6 hours.

Supplementary Figure 2 PLS-DA analysis was performed on data collected by positive ion mode as in comparison with the negative ion mode.

Supplementary Figure 3 Invasion and EMT studies.These studies were done the same way as described for . **(A)** Invasion rate study in A549 cells. **(B)** Invasion rate study in H1299 cells. **(C)** Quantification of the western blot analysis.

Supplementary Figure 4 Quantification of filopodia induced by either eATP or TGF-β at different induction times. A549 and H1299 human NSCLC cells were treated by either eATP or TGF-β for 2, 6, and 12 hours. The formation of filopodia were photographed by fluorescence microscopy and counted from >10 randomly chosen views for each condition (average 100-200 cells per condition). Filopodia growing on the cells were arbitrarily categorized as none, early, mid, and late stages, based on their size, shape and number, and were separately counted by four individuals and then averaged. **(A)** A549 cells. **(B)** H1299 cells. These results indicate that eATP induced time-dependent filopodia formation similarly to TGF-β.

Supplementary Figure 5 Intracellular ATP measurement study. This study was done the same way as described for but on H1299 cells. **(A)** iATP levels of H1299 cells treated with different concentrations of eATP. **(B)** iATP levels of H1299 cells treated with different concentrations of TGF-β. Untreated cells were used as negative control.

Supplementary Figure 6 Quantification of the soft-agar colony formation assay. This quantification is for the assay shown in Figure 8G.

Supplementary Table 1 Epithelial genes significantly changed by either ATP or TGF-β treatment.

Supplementary Table 2 FPKM values of three replicate samples for each gene for heatmap in **Figure 1G**.

Supplementary Table 3 FPKM values of three replicate samples for each gene for heatmap in **Figure 1H**.

Supplementary Table 4 FPKM values of three replicate samples for each gene for heatmap of **Figure 4D**.

Supplementary Table 5 FPKM values of three replicate samples for each gene for heatmap of **Figure 5E**.

Supplementary Table 6 FPKM values of three replicate samples for each gene for heatmap of **Figure 5F**.

## Abbreviations

EMT, Epithelial mesenchymal transition; eATP, Extracellular ATP; GSEA, Gene set enrichment analysis; ieATP, Intratumoral eATP; iATP, Intracellular ATP; TGF- β, Transforming growth factor beta; NES, Normalized enrichment score; NSCLC, Non-small cell lung carcinoma; PR, Purinergic receptor; TME, Tumor microenvironment; DMEM, Dulbecco’s Modified Eagle Medium; PLS-DA, Partial least squares-discriminant analysis; Log2fc, Log2 fold change.
