# Supplementary material for: From Transcriptomics, Metabolomics to Functional Studies: Extracellular ATP Induces TGF-β-Like Epithelial Mesenchymal Transition in Lung Cancer Cells
Source: Front Oncol. 2022 Jun 30;12:912065. doi: 10.3389/fonc.2022.912065 (PMC9282887; doi:10.3389/fonc.2022.912065)
Supplement: Supplementary Figure 1 — GSEA plots of gene enrichment in cells treated with either eATP or TGF-β. GSEA plots were made the same way as and . (A) Downregulated genes. (B) Downregulated genes involved in or not known to be involved in EMT. (C) Gene enrichment at 2 hours. (D) Gene enrichment at 6 hours. [file DataSheet_1.docx]

**Supplemental Figures**

**Fig. S1**

**a. b.**


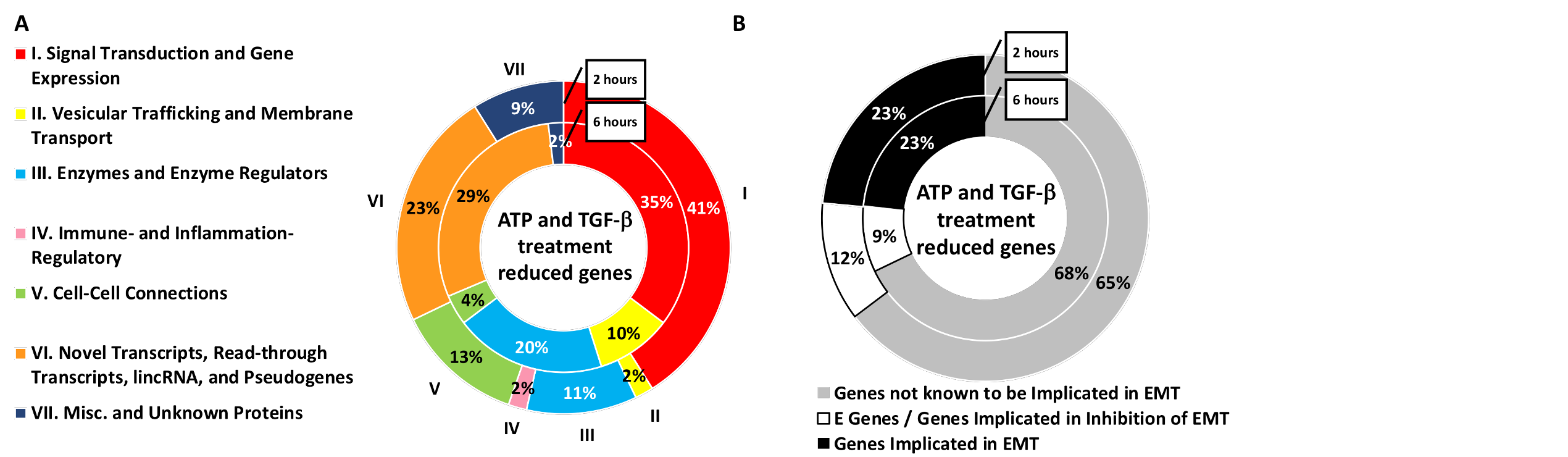


**c.**


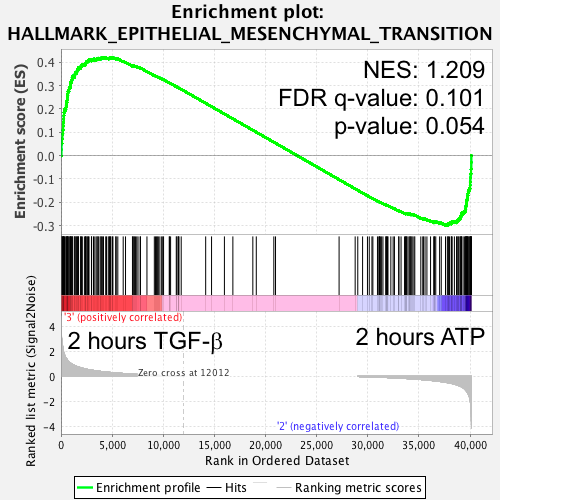


**d.**


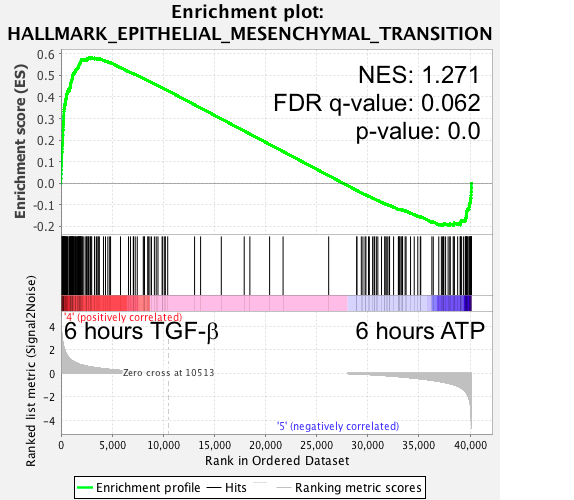


**Fig. S2**


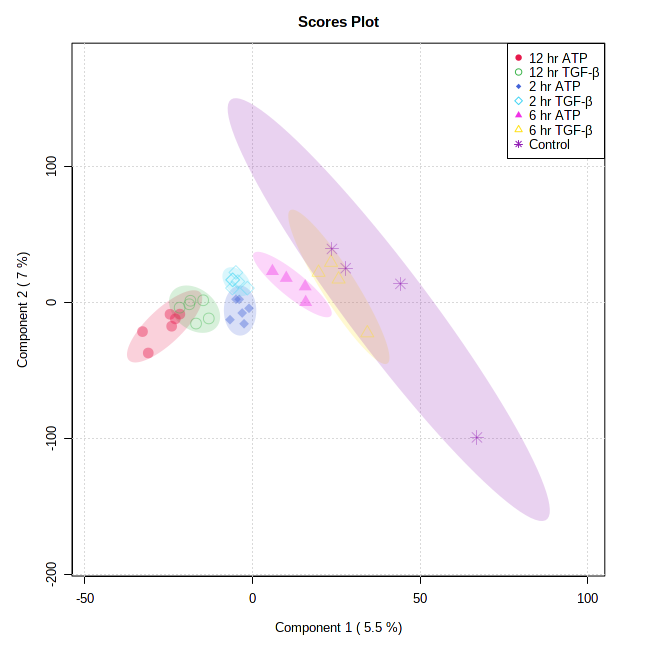


**Fig. S3**

**a**.


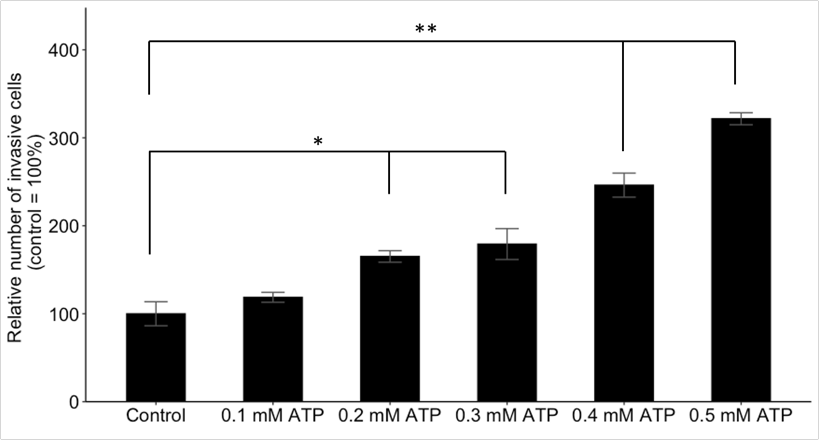


**b.**


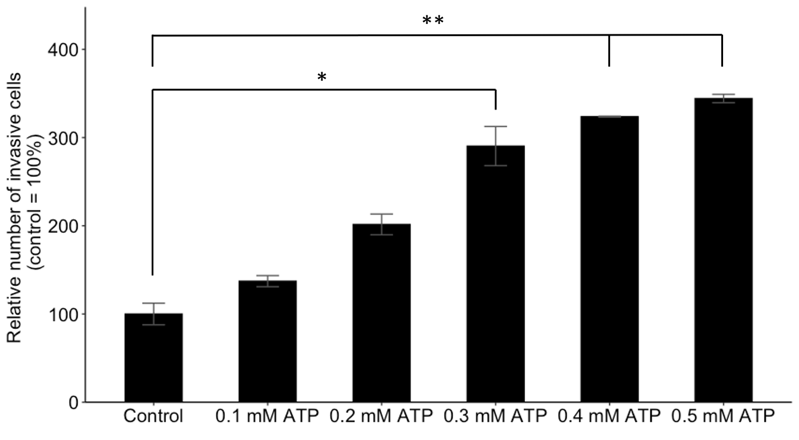


**c.**


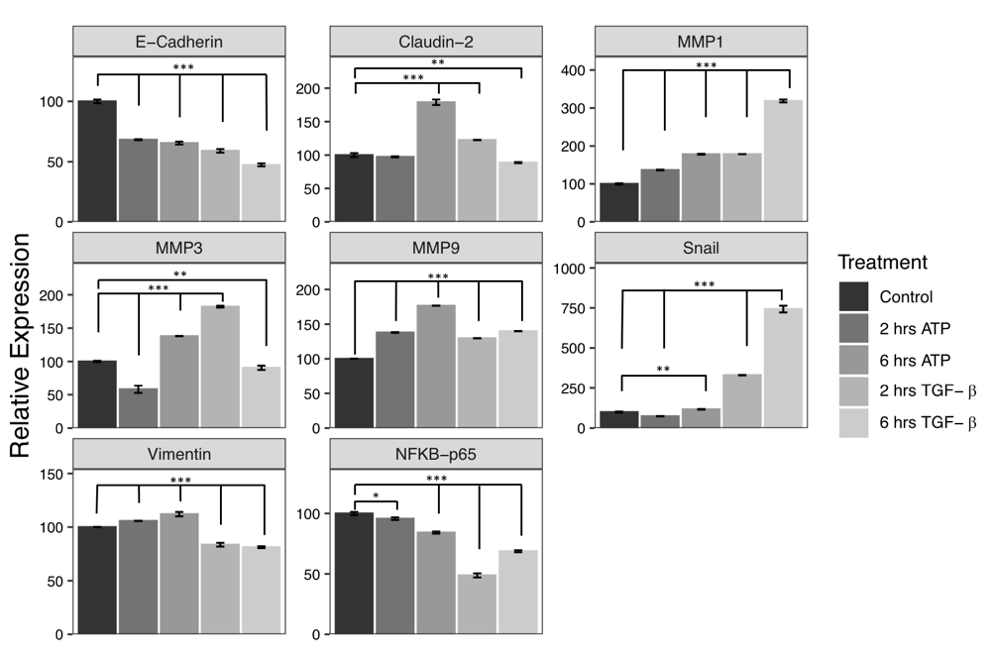


**a.**

**b.**


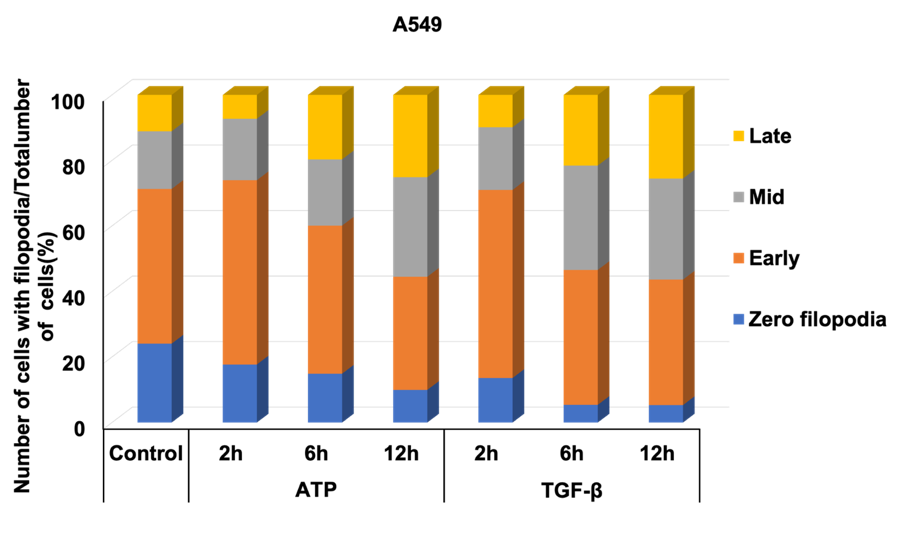


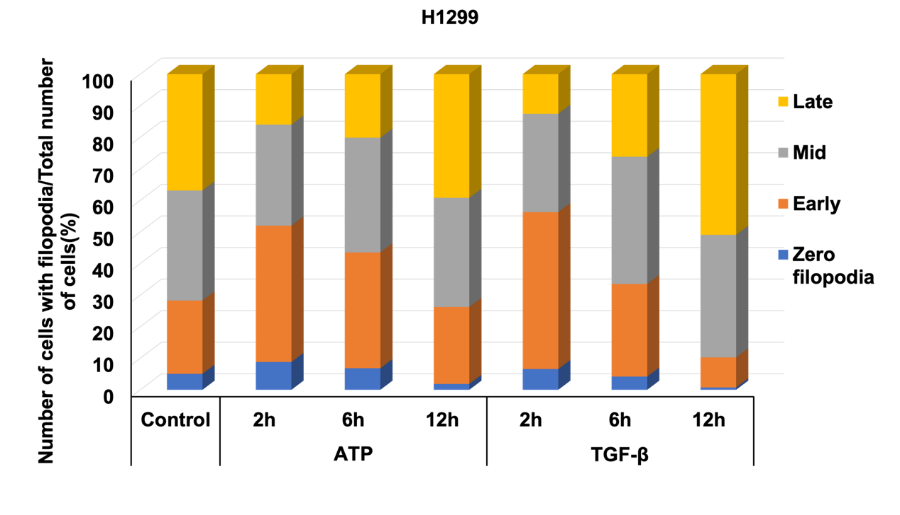


**Fig. S4**

**Fig. S5**

**a.**


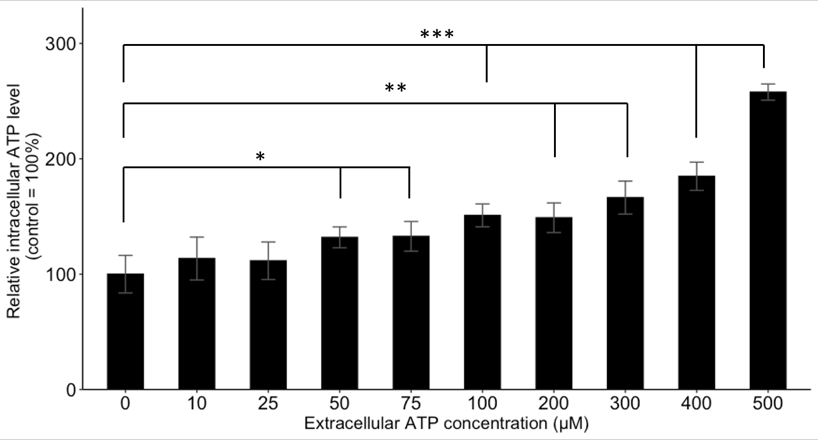


**b.**


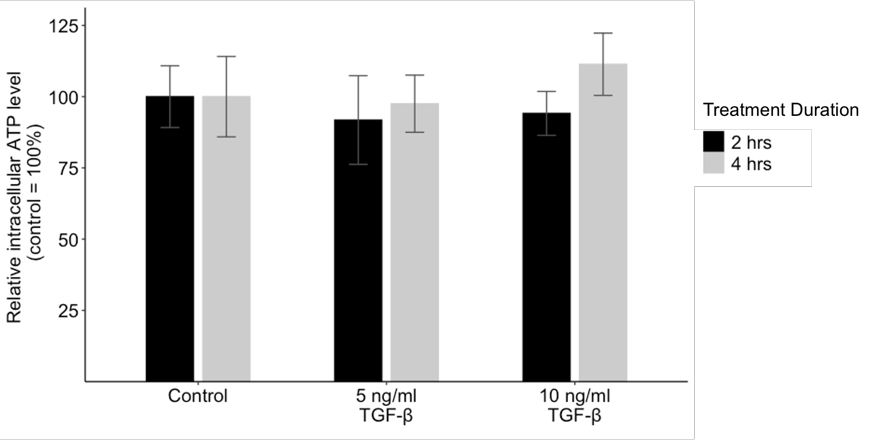


**FIG S6**


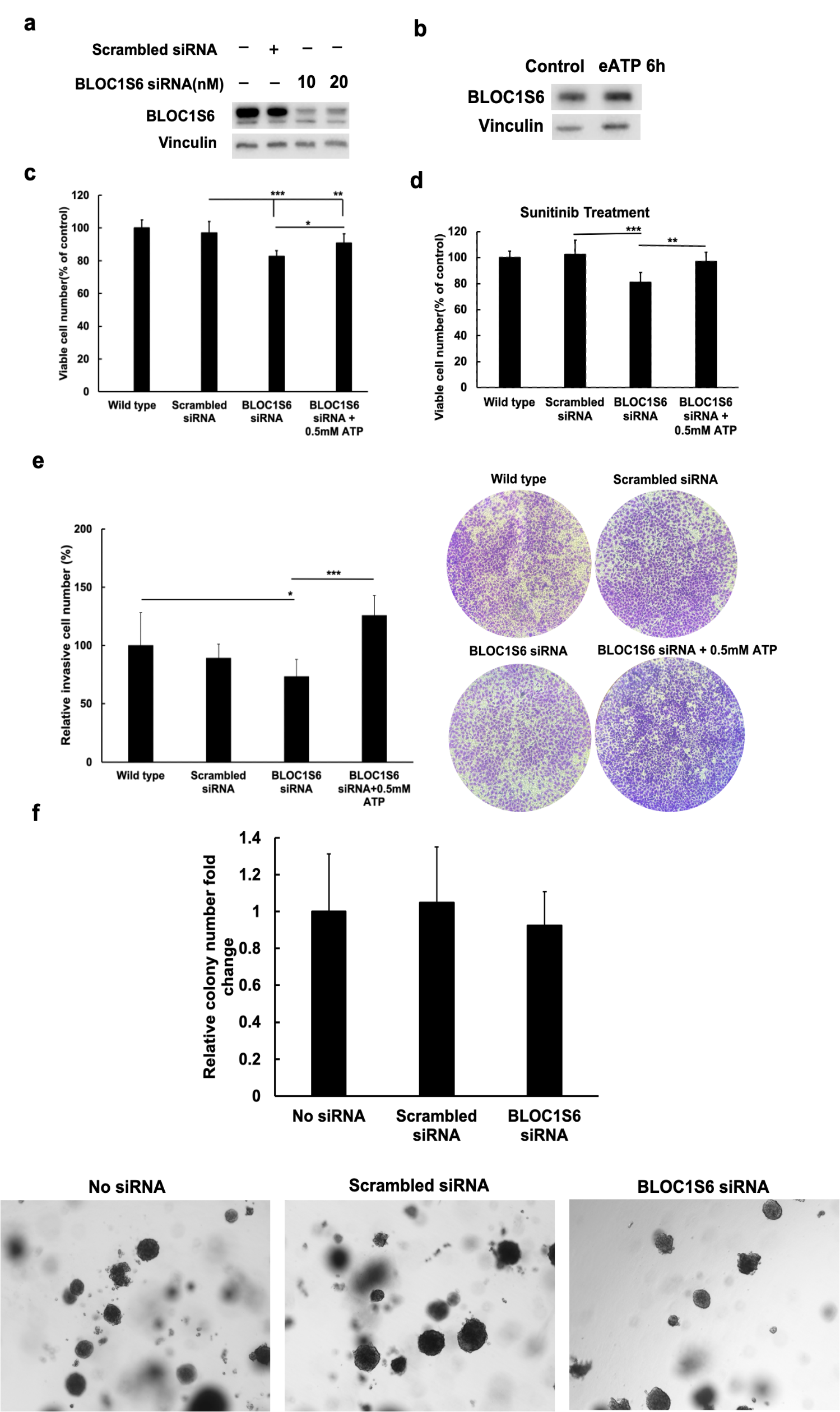


**Supplemental Table 1**


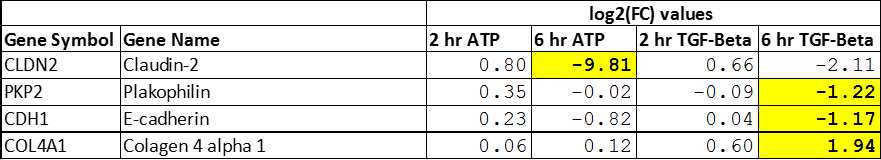

**Supplemental Table 2**

**Supplemental Table 3**

**Supplemental Table 4**

**Supplemental Table 5**

**Supplemental Table 6**
